# Draft Genome Sequence of *Bradyrhizobium japonicum* Is-34, Which Is Incompatible with *Rj4* Genotype Soybeans

**DOI:** 10.1128/genomeA.01316-14

**Published:** 2014-12-18

**Authors:** Hirohito Tsurumaru, Yu Kanesaki, Syougo Hashimoto, Kouhei Okizaki, Hirofumi Yoshikawa, Takeo Yamakawa

**Affiliations:** a Graduate School of Life Science, Tohoku University, Sendai, Japan; b Department of Biosciences and Biotechnology, Faculty of Agriculture, Kyushu University, Fukuoka, Japan; c Genome Research Center, NODAI Research Institute, Tokyo University of Agriculture, Tokyo, Japan; d Graduate School of Bioresource and Bioenvironmental Sciences, Faculty of Agriculture, Kyushu University, Fukuoka, Japan; e Department of Bioscience, Tokyo University of Agriculture, Tokyo, Japan

## Abstract

We report here the draft genome sequence of *Bradyrhizobium japonicum* Is-34, which is incompatible with *Rj4* genotype soybeans. A candidate gene involved in this incompatibility was found to be present in this genome.

## GENOME ANNOUNCEMENT

*Bradyrhizobium* bacteria establish a symbiosis with soybeans (*Glycine max*) by forming root nodules. In the root nodules, *Bradyrhizobium* bacteria provide fixed nitrogen to the soybean, and in return acquire carbohydrates from the soybean. However, some soybean cultivars restrict nodulation of specific strains such as *B. japonicum* Is-34 and *B. elkanii* USDA61 (1). This restriction is due to the *Rj4* gene in soybeans, and recent studies have revealed that the *Rj4* gene encodes a thaumatin-like protein (1,2). In contrast, a causal gene in the incompatible *Bradyrhizobium* strains has not been identified.

Genomic DNA of *B. japonicum* Is-34 was extracted with the ISOPLANT kit (Nippon Gene, Tokyo, Japan), and fragmented to 400 bp using the Covaris S2-A system (Covaris, Woburn, MA, USA). A library for sequencing was prepared by a NEBNext DNA library prep master mix set for Illumina (New England Biolabs, Ipswich, MA, USA), and paired-end sequenced (2 × 300 bp) on a MiSeq sequencer using a MiSeq version 3 reagent kit (Illumina KK, Tokyo, Japan). Raw reads were trimmed and *de novo* assembled using the CLC Genomics Workbench version 7.5 (Qiagen, Valencia, CA, USA). Parameters for the trimming were as follows: ambiguous limit, 2; quality limit, 0.001; number of 5′ terminal nucleotides, 10; and number of 3′ terminal nucleotides, 40. Parameters for the *de novo* assembly were as follows: update contigs, yes; bubble size, 600; minimum contig length, 1,000; word size, 51; perform scaffolding, yes; auto-detect paired distances, yes; mismatch cost, 2; insertion cost, 3; deletion cost, 3; length fraction, 0.5; and similarity fraction, 0.8.

The draft genome of *B. japonicum* Is-34 was assembled into 248 contigs, with an accumulated length of 10,326,597 bp (*N*_50_ = 472,902 bp) and an average GC content of 63.1%. The genome was annotated by the NCBI Prokaryotic Genome Annotation Pipeline (PGAP version 2.8), and a total of 9,145 coding sequences (CDSs), 6 rRNAs, and 69 tRNAs were predicted.

As a candidate gene for the incompatibility with *Rj4* genotype soybeans, an effector protein of type III secretion system (T3SS) was postulated because disruptants of the T3SS structural genes in the incompatible *Bradyrhizobium* strain overcame the nodulation restriction of *Rj4* genotype soybeans (3). In the present report, a candidate effector gene (MA20_12780) was found in the genome of *B. japonicum* Is-34. A tts box motif (3) was present in the upstream of this gene, indicating that this gene was a type III-secreted protein. An NCBI conserved domain search (http://www.ncbi.nlm.nih.gov/Structure/cdd/wrpsb.cgi) revealed that this gene belongs to the C48 peptidase (the ubiquitin-like protease 1; Ulp1) family. In addition, the candidate gene (MA20_12780)-disrupted mutant of *B. japonicum* Is-34 overcame the nodulation restriction of *Rj4* genotype soybeans. Detailed characterization of this disruptant will be reported in the near future.

### Nucleotide sequence accession numbers.

This whole-genome shotgun project has been deposited at DDBJ/EMBL/GenBank under the accession no. JRPN00000000. The version described in this paper is version JRPN01000000.
